# The prevalence and associated factors of hepatitis B and C virus in hemodialysis patients in Africa: A systematic review and meta-analysis

**DOI:** 10.1371/journal.pone.0251570

**Published:** 2021-06-22

**Authors:** Tiruneh Adane, Solomon Getawa

**Affiliations:** Department of Hematology and Immunohematology, School of Biomedical and Laboratory Sciences, College of Medicine and Health Sciences, University of Gondar, Gondar, Ethiopia; Centre de Recherche en Cancerologie de Lyon, FRANCE

## Abstract

**Background:**

Due to its invasive procedure patients on hemodialysis (HD) are at high risk of infections. Infections acquired in dialysis units can prolong hospitalization date and/or prolong illness in patients, and increase treatment cost. There are no adequate data on the prevalence of Hepatitis B virus (HBV) and Hepatitis C virus (HCV) infections in HD patients. Therefore, this study aimed to estimate the pooled prevalence and associated factors of HBV and HCV infections among HD patients in Africa.

**Method:**

The databases PubMed, Medline, EMBASE, Cochrane library, web of science, African Journals Online, Science Direct, and Google Scholar were searched to identify relevant studies. The review was performed based on Preferred Reporting Items for Systematic Reviews and Meta-Analyses (PRISMA) guidelines. Data were extracted independently by two authors and analyzed using STATA 11. A random-effect model was fitted to estimate the pooled prevalence with their 95% confidence interval. To detect publication bias funnel plots analysis and Egger weighted regression tests were done.

**Results:**

The overall pooled prevalence of HBV and HCV infection among HD patients in Africa was 9.88% (95% CI: 7.20–12.56) I^2^ = 97.9% and 23.04% (95% CI: 18.51–2757) I^2^ = 99.6%, respectively. In addition, the pooled prevalence of HBV and HCV co-infection was 7.18% (95% CI: 3.15–11.20) I^2^ = 99.6%. Duration of dialysis was found to be the contributing factor for the occurrence of HBV and HCV among HD patients (OR = 1.44; 95% CI: 1.04, 2.01).

**Conclusion:**

This study showed that there is high prevalence of HBV and HCV infections in HD patients in Africa. Therefore, strict adherence to precautions of infection control measures, isolation of seropositive patients, improvement in infrastructures, adequate screening of HBV and HCV for the donated blood, and decentralized HD services is needed to minimize the risk of HBV and HCV infections in HD facilities.

## Introduction

Hepatitis is inflammation of the liver due to viral infection and leads to significant morbidity and mortality [1]. The HBV and HCV infection are highly infectious and transmitted from person to person by blood transfusions, sexual, and vertical routes [2]. They are the most common viral infections among individuals with renal disease [3]. Patents having renal disease do not clear the viral infections efficiently, hence the disease is associated with reduced immunity [4]. Patients with end‑stage renal disease (ESRD) are at increased risk of acquiring HBV and HCV infections than the general population due to their deficient immune response, exposure to blood transfusions, and HD equipment [5]. These patients are often anemic, require prolonged vascular access, and have a high possibility of exposure to infected patients and contaminated equipment, and cross-contamination from the dialysis circuits [6]. Nosocomial and iatrogenic transmission, duration of ESRD, duration of dialysis, and mode of dialysis are other risk factors in HD patients [7,8]. HD patients are potentially susceptible to infection with these blood-borne viral agents (HBV and HCV) compared to the general population making them a persistent public health concern as they are a cause of increased morbidity and mortality [9,10].

Africa has the second largest number of chronic HBV carriers after Asia and is considered as a region of high endemicity [11]. HCV is highly prevalent in patients undergoing maintenance HD where it adversely affects patient survival [12,13]. The global prevalence of HCV infection ranges from 0.6% in Canada, 1.5% in Japan, to 6% in Africa [14]. The prevalence of HBV and HCV during HD varies across countries. Strict infection control measures implemented in developed countries has minimized the transmission rate. However, the prevalence remains high in developing countries [15]. This might be due to low strict adherence to standard precautions and routine HD precautions, the absence of vaccinations, and the lack of financial resources [16].

There were a lot of studies that reported the prevalence of HBV and HCV among HD patients in different countries of Africa. However, there was no study that showed the pooled prevalence and associated factors of HBV and HCV among HD patients in the continent. Therefore, this systematic review and meta-analysis aimed to estimate the pooled prevalence and associated factors of HBV and HCV in HD patients in Africa. The study also determines the pooled prevalence of HBV and HCV virus in those patients in the different geographical areas (Northern, Central, Western, Eastern, and Southern) of Africa. The result of this study may provide insight to public health policymakers to plan appropriate public health interventions in the continent and also national level.

## Methods and materials

### Study design and protocol registration

This systematic review and meta-analysis were performed per the PRISMA guideline [17] (S1 Table). The protocol has been registered in the International Prospective Register of Systematic Reviews (PROSPERO), with the assigned number of CRD42021224905.

### Search strategy

PubMed, Medline, EMBASE, Cochrane library, web of science, African Journals Online, Science Direct, and Google Scholar were the databases searched by two authors (TA and SG) to identify all articles reporting the prevalence of HBV and HCV infection in HD patients in Africa. Besides, a manual search was also conducted for relevant articles. We used the search terms separately and in combination using Boolean operators like “OR” or “AND”. An example of a search strategy used was as follows: ((((((((((prevalence) AND (seroprevalence)) OR (hepatitis)) OR (hepatitis viruses)) OR (hepatitis B virus)) OR (hepatitis C virus)) OR (HBV)) OR (HCV)) AND (hemodialysis patients)) OR (HD patients)) AND using African search filter developed by Pienaar et al to identify prevalence studies [18].

### Eligibility criteria

#### Inclusion criteria

Cross-sectional and cohort studies which reported the prevalence of HBV and/or HCV infection, published in peer-reviewed journals in English language only, conducted in the continent of Africa, used the HBsAg test to diagnose HBV infection and anti-HCV antibody test for HCV infection, and published from 1998 to 30 November 2020 were included in this review.

#### Exclusion criteria

Studies were excluded if they did not report the prevalence of HBV and/or HCV, if they were case reports, reviews, poster presentations, and editorials letters, if they were published in non-English languages, and were conducted outside the continent of Africa.

### Study selection and quality assessment

After removing ineligible or duplicated papers, all potential eligible papers were reviewed. Full-text papers were retrieved for review and extraction of relevant information. The authors agreed to settle their disagreement through discussion. The Joana Brigg’s Institute (JBI) critical appraisal checklist for simple prevalence was used to assess the quality of included studies [19]. This tool comprised of 9 questions. (1) Was the sample frame appropriate to address the target population? (2) Were study participants sampled appropriately? (3) Was the sample size adequate? (4) Were the study subjects and the setting described in detail? (5) Was the data analysis conducted with sufficient coverage of the identified sample? (6) Were valid methods used for the identification of the condition? (7) Was the condition measured in a standard, reliable way for all participants? (8) Was there an appropriate statistical analysis? (9) Was the response rate adequate? For each question, a score of 0 was assigned for ‘not reported or not appropriate’ and 1 for ‘yes’. Then, the scores were summarized to get a total score ranging from 0 to 9. Based on the assigned points, articles were categorized as having a high (7–9), medium (5–7), or low (0–4) quality. Those articles with high and medium quality were included in the final analysis (S2 Table).

### Data extraction

Two authors (SG and TA) extracted the necessary information from all the included studies including the name of the author, publication year, study year, the mean age of study participants, type of study, the sample size, sampling techniques, number of the infected cases, duration of dialysis, and methods used to detect HBsAg or anti-HCV antibody.

### Statistical analysis

Eligible data were extracted and entered into Microsoft Excel and then exported to Stata version 11.0 software for analysis. The pooled prevalence of HBV and HCV along with the 95% confidence intervals was visually displayed using a forest plot. The heterogeneity of the included studies was evaluated using an index of heterogeneity (I^2^ statistic). Low, moderate, and high levels of heterogeneity were considered when the values of I^2^ becomes 25%, 50%, and 75%, respectively [20]. In all the pooled analyses, a random-effects model was used when heterogeneity was present due to variations of effects from individual studies. The potential sources of heterogeneity were identified using sub-group, meta-regression, and sensitivity analysis. Publication bias was investigated statistically using Egger’s test. A p-value < 0.05 in Egger’s test was considered as evidence of statistically significant publication bias.

## Results

**Fig 1:**
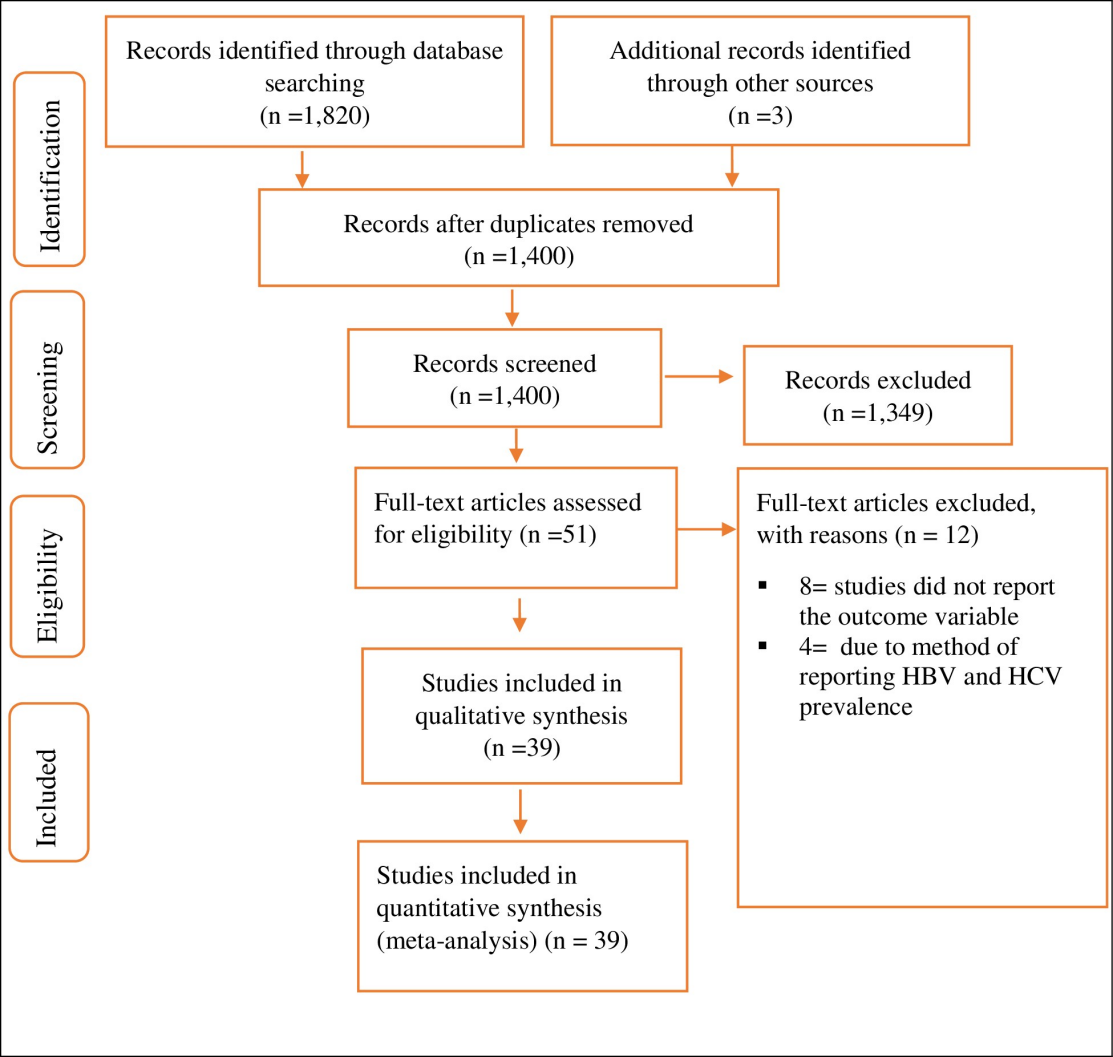
Flow chart to describe the selection of studies for the systematic review and meta-analysis on the prevalence of HBV and HCV infection among HD patients.

Of 1,820 studies identified in the initial search, 423 were removed due to duplicates. Moreover, 1,349 studies were excluded in the title and abstract screening, and in the full‑text screening, 23 cases were excluded. Finally, all the 39 studies were included in the final meta-analysis (Fig 1).

### Characteristics of included studies

A total of 39 published articles on 23,538 HD patients were included in this review. Regarding the study design, 24 and 5 studies had used a cross-sectional and retrospective design, respectively. However, the other 10 studies did not report their research design. Of the total studies, 5 studies had used a convenient sampling while the other 34 studies did not report their sampling techniques. Of the 39 studies included, five studies were conducted in Nigeria, 3 in Cameroon, 5 in Sudan, 5 in Libya, 8 in Egypt, 1 in Senegal, 1 in Angola, 1 in Togo, 4 in Tunisia, 1 in Ethiopia, 1 in South Africa, 3 in Morocco, and 1 in Kenya. The minimum and maximum numbers of study participants were 46 in Sudan [21] and 4290 in Tunisia [22], respectively. The mean age of study participants ranged from 39.9 to 53.4 years. Of the 39 studies, 19 studies reported the prevalence of both HBV and HCV, 16 studies reported HCV alone, and 4 studies reported HBV alone. Sixteen studies reported the average duration of dialysis of the patients. Twenty-six and 5 studies reported the HBV and/or HCV prevalence using ELISA (enzyme-linked immunosorbent assay) and rapid kit tests, respectively. However, 5 studies did not explain the type of test they used for the determination of hepatitis infection. Reverse passive hemagglutination (RPHA), CILA (chemiluminescence immunoassay), and microparticle enzyme immunoassay (MEIA) tests were used by 3 separate studies (Table 1).

**Table 1:** Description and outcomes of included studies.

Author, year of Publication	Country	Study Year	Study design	Sample Size	Sampling method	Cases	Mean age (years)	Mean HD duration	HBV (%)	HCV (%)	Coinfec (%)	Method
Okoye OG,2020 [23]	Nigeria	2012–2016	cross-sectional	341	NR	15	NR	1mon	2.6	2.3	-	rapid kit
Chizoba et al,2018 [24]	Nigeria	2016	cross-sectional	90	NR	10	NR	NA	4.4	6.7	-	rapid kit
Ali et al,2017 [25]	Libya	2013–2016	cross-sectional	645	NR	69	NR	NA	0.8	9.92	-	ELISA
Gasim et al,2012 [26]	Sudan	2010	cross-sectional	353	NR	46	46.24 ±15.3	30.3(26.8)m	4.5	8.5	-	ELISA
Hammad et al,2016 [27]	Sudan	2015	cross-sectional	100	Convenient	11	50(20–80)	3,5 years	5	6	-	ELISA
Gusbi et al,2019 [28]	Libya	NR	cross-sectional	2325	NR	388	53.40±15	NR	4	16.7	8.7	ELISA
Mhalla et al,2017 [29]	Tunisia	2013–2014	cross-sectional	109	NR	14	50 ± 14.7	NR	5.5	7.3	-	CLIA
Luma et al,2017 [30]	Cameroon	2013	Cross-sectional	104	NR	31	48 ±16	14 m	10.6	19.2	-	ELISA
El-Amin et al,2007 [31]	Sudan	2005	Cross-sectional	236	NR	76	43.6 ± 15.6	36.6 ±35.1m	15.9	23.7	-	ELISA
Alashek et al, 2012 [32]	Libya	2009–2010	Cross-sectional	2382	NR		49 (36–61)	NR	2.6	31.1	38	ELISA
Otedo et al 2003 [33]	Kenya	1998	Cross-sectional	100	NR	13	44.3± 14.6	1.74 ± 0.9	8	5	-	RPHA
Juhar et al 2018 [34]	Ethiopia	2016	Cross-sectional	253	NR	10	48.94 ± 16	NR	1.2	2.8	0.4	ELISA
Amira et al 2020 [35]	Nigeria	1996–2012	Retrospective	1388	NR	99	46.1 ± 15.3	NR	6.0	1.2	0.1	NR
Sarhan et 2015 [36]	Egypt	2011	Retrospective cross-sectional	987	NR	447	NR	3.35±2.048	45.2	0.1	0.6	NR
Lioussfi, et al 2014 [37]	Morocco	2009	Retrospective cross-sectional	67	NR	44	44.2 ± 12	10.6 ± 5.2	6	60		ELISA
Zeinab et al 1994 [38]	Egypt	NR	NR	134	NR	NR	NR	NR	9.4	89	-	NR
Abdelaali et al, 2013 [39]	Morocco	2002–2010	NR	163	NR	78	51.25 ± 14.9	6 years	8	39.9	1.8 co	ELISA
Eljamay, 2019 [40]	Libya	2012–2013	cross- sectional	62	NR	3	NR	NR	1.6	3.2		ELISA
Halle et al,2016 [41]	Cameroon	2012	cross-sectional	97	NR	26	51 ± 14	32.8 ± 27.5	6.2	20.6	2.1	ELISA
Cassidy et al 1995 [42]	South Africa	NR	NR	103	NR	22	44 (23–79)	75 (3–196)	-	21	-	ELISA
Senosy et al, 2016 [43]	Egypt	2015	Retrospective cross-sectional	971	NR	591	46.14±9.9	5.4±2.3	-	60.9	-	NR
Seck et al 2014 [44]	Senegal	2011	Cross-sectional	106	Convenient	6	43.4 ± 15.8	60.5 ± 15 m	-	5.6	-	ELISA
Ummate et al,2013 [45]	Nigeria	NR	cross-sectional	100	NR	15	39.9± 13.58	NR	-	15	-	ELISA
Elzouki et al 1995 [46]	Libya	NR	Cross-sectional	153	NR	32	41	NR		21	6	ELISA
Hmaied et al 2006 [47]	Tunisia	2001–2003	NR	395	NR	79	54	NR		20		ELISA
Sassi et al, 2000 [48]	Tunisia	NR	NR	58	NR	27	16–77	NR	-	46.5	-	ELISA
Ayed et al, 2003 [22]	Tunisia	2001	NR	4290	NR	828	NR	NR	-	19.25		ELISA
Suliman et al, 1995 [21]	Sudan	1994	NR	46	NR	16	NR	3.28		34.9		ELISA
Khodir et al 2012 [49]	Egypt	2001	NR	2351	NR	992	52±11	NR		35		MEIA
Borges et al,2018 [50]	Angola	2016	cross‐sectional	1075	NR	60	NR	N	-	5.6	-	rapid kit
Ibrahim et al, 2013 [51]	Egypt	2014–2016	NR	90	NR	44	54.41±10.81	NR	-	48.9	-	ELISA
Foullous et al,2015 [52]	Morocco	NR	cross-sectional	630	NR	194	48.33±15.5	NR	-	30.79	-	ELISA
Zahran, 2014 [53]	Egypt	NR	Retrospective	514	NR	255	NR	NR	-	49.6	-	NR
Samah et al,2015 [54]	Sudan	2010	Cross-sectional	308	Convenient	44	NR	NR	-	14.3	-	ELISA
Elzorkany et al,2017 [55]	Egypt	2016	cross-sectional	1891	NR	719	53.16±13.34	NR	-	41.9	-	ELISA
Salou et al,2019 [56]	Togo	2016	cross-sectional	95	Convenient	10	46.6(13–80)	51.7 months	10.5	-	-	rapid kit
Nkup et al 2017 [57]	Nigeria	NR	Cross-sectional	110	NR	17	NR	NR	15.5	-	-	Rapid kit
Halle et al,2013 [58]	Cameroon	2012	cross-sectional	166	Convenient	13	49.2 ± 14.2	24m (8–42)	7.8	-	-	ELISA
Maksoud et al 2019 [59]	Egypt	2015–2016	NR	150	NR		46.7 ± 11.45	NR	60.7	-	-	ELISA

NA; not appropriate, NR; not reported.

### Prevalence of HBV and HCV

Thirty-nine published studies were included in this systematic review and meta-analysis to estimate the pooled prevalence of HBV and HCV infection among HD patients. The minimum prevalence was 0.8% (Libya) [25] and 2.3% (Nigeria) [23] for HBV and HCV, respectively. On the other hand, the maximum prevalence of HBV and HCV was 60.7% (Egypt) [59] and 60.9% (Egypt) [43], respectively. A total of 23 studies reported the prevalence of HBV in HD patients. Accordingly, the pooled prevalence of HBV was 9.88% (95% CI: 7.20–12.56) I^2^ = 97.9% (Table 2).

**Table 2:** Prevalence of HBV among HD patients in Africa.

Characteristics	Studies	sample	cases	Prevalence (95%CI)	I^2^ (%)	p-value	Egger test
Prevalence of HCV in HD patients	23	10,457	971	9.88(7.20–11.56)	97.9	< 0.001	0.013
**Study year**							
1996–2008	2	336	28	12.06(4.32–19.80)	79.1	0.029	
2009–2015	12	5,978	686	9.09(3.76–14.43)	98.5	< 0.001	
2016–2020	6	1,574	135	11.17 (5.89–16.45)	97.9	< 0.001	
N/A	3	2,569	122	8.97(2.41–15.53)	86.8	0.001	
**Diagnostic method**							
Rapid test kit	4	636	46	7.50 (2.26–12.73)	83.4	< 0.001	
ELISA	14	7,103	370	7.59 (5.38–9.80)	95.9	< 0.001	
Others	2	209	14	6.48(3.15–9.82)	0.0	0.473	
N/A	3	2,509	541	20.21(-6.51–46.92)	99.6	< 0.001	
**Region**							
Northern Africa	13	7,713	774	12.27 (8.19–16.35)	98.8	< 0.001	
Central Africa	3	367	50	7.88(5.13–10.63)	0.0	0.526	
Western Africa	5	2,024	129	6.51(3.48–9.54)	82.3	< 0.001	
East Africa	2	353	18	4.09(-2.50–10.68)	83.1	0.015	

On the other hand, a meta-analysis of 35 studies for HCV showed that the pooled prevalence of HCV in HD patients was and 23.04% (95% CI: 18.51–2757) I^2^ = 99.6% (Table 3). Moreover, the pooled prevalence of HBV and HCV coinfection was 7.18% (95% CI: 3.15–11.20) I^2^ = 99.6%.

**Table 3:** Prevalence of HCV among HD patients in Africa.

Characteristics	Studies	sample	cases	Prevalence (95%CI)	I^2^ (%)	p-value	Egger test
Prevalence of HCV in HD patients	35	23,017	5,542	23.04(18.51–27.57)	99.6	*<* 0.001	0.000
**Study year**							
1994–2008	6	7,418	1,976	22.42(13.83–31.02)	98.1	*<* 0.001	
2009–2015	14	7,197	1,596	19.07(13.44–24.69)	99.5	*<* 0.001	
2016–2020	7	4,385	918	16.39(5.45–27.32)	99.4	*<* 0.001	
N/A	8	4,017	1,052	36.16(19.25–53.08)	99.2	*<* 0.001	
**Diagnostic method**							
Rapid test kit	3	1,506	81	4.46(1.69–7.23)	80.6	0.006	
ELISA	24	14,957	3,474	22.55 (17.76–27.34)	98.1	*<* 0.001	
Others	3	2,560	1,005	15.84 (-6.45–38.13)	99.1	*<* 0.001	
N/A	5	3,994	982	39.52(29.13–49.92)	99.9	*<* 0.001	
**Region**							
Northern Africa	24	19,260	5,341	29.69(21.47–37.91)	99.7	*<* 0.001	
Central Africa	3	1,276	106	14.63(3.18–26.07)	91.7	*<* 0.001	
Western Africa	5	2,025	58	4.37(1.75–7.00)	83.0	*<* 0.001	
East Africa	2	353	15	3.21(1.37–5.04)	0.0	0.362	
South Africa	1	103	22	21.00)13.13–28.87)	-	-	

### Subgroup analysis of detection method, region, and year of study

The prevalence of HBV among different types of detection methods, regions of the studies performed, and years of the study were analyzed by subgroup analysis. The combined prevalence of HBV by ELISA, rapid kit, other methods, and NA (methods not clear) was 7.59 (95%CI: 5.38–9.80), 7.50% (95% CI: 2.26–12.73), 6.48% (95% CI: 3.15–9.82), and 20.21% (95% CI: -6.51–49.92), respectively. The combined prevalence of HBV in Western, Central, Northern, and East African countries was 6.57% (95% CI: 3.48–9.58), 7.88% (95% CI: 5.13–10.63), 12.27% (95% CI: 8.19–16.35), and 4.09% (95% CI: -2.50–10.68), respectively. The pooled prevalence of HBV per year of study was as follows: 1994–2008; 11.22% (95% CI: 6.34–16.09), 2009–2015; 5.10% (95% CI: 2.54–7.66), 2016–2020; 11.70% (95% CI: 6.79–16.61), and NA (study year not clear); 4.0% (955 CI: 3.20–4.80) (Table 2).

Across regions, the combined prevalence of HCV among HD patients was as follows: Northern Africa 29.69% (95% CI: 21.47–37.91), Central Africa, 14.63% (95% CI: 3.18–26.07), Western Africa, 4.37% (95% CI: 1.75–7.00), South Africa, 21.00% (95% CI: 13.13–28.87), and East Africa, 3.21% (95% CI: 1.37–5.04). Furthermore, the pooled prevalence of HCV in HD patients was 12.66% (95% CI: -4.32–19.80), 9.09% (95%CI: 3.76–14.43), 11.17% (95% CI: 5.89–16.45), and 8.97% (95% CI: 2.41–15.53) in 1996–2008, 2009–2015, 2016–2020, and NA (year of study not clear, respectively. The combined prevalence of HCV in HD patients was 22.55% (95% CI: 17.76–27.34), 4.46% (95% CI: 1.89–7.23), 15.84% (95% CI: 6.45–38.13), 39.52% (95% CI: -29.13–49.92) according to ELISA, rapid kit, other methods, and NA (methods not clear) (Table 3).

#### Publication bias

**Fig 2:**
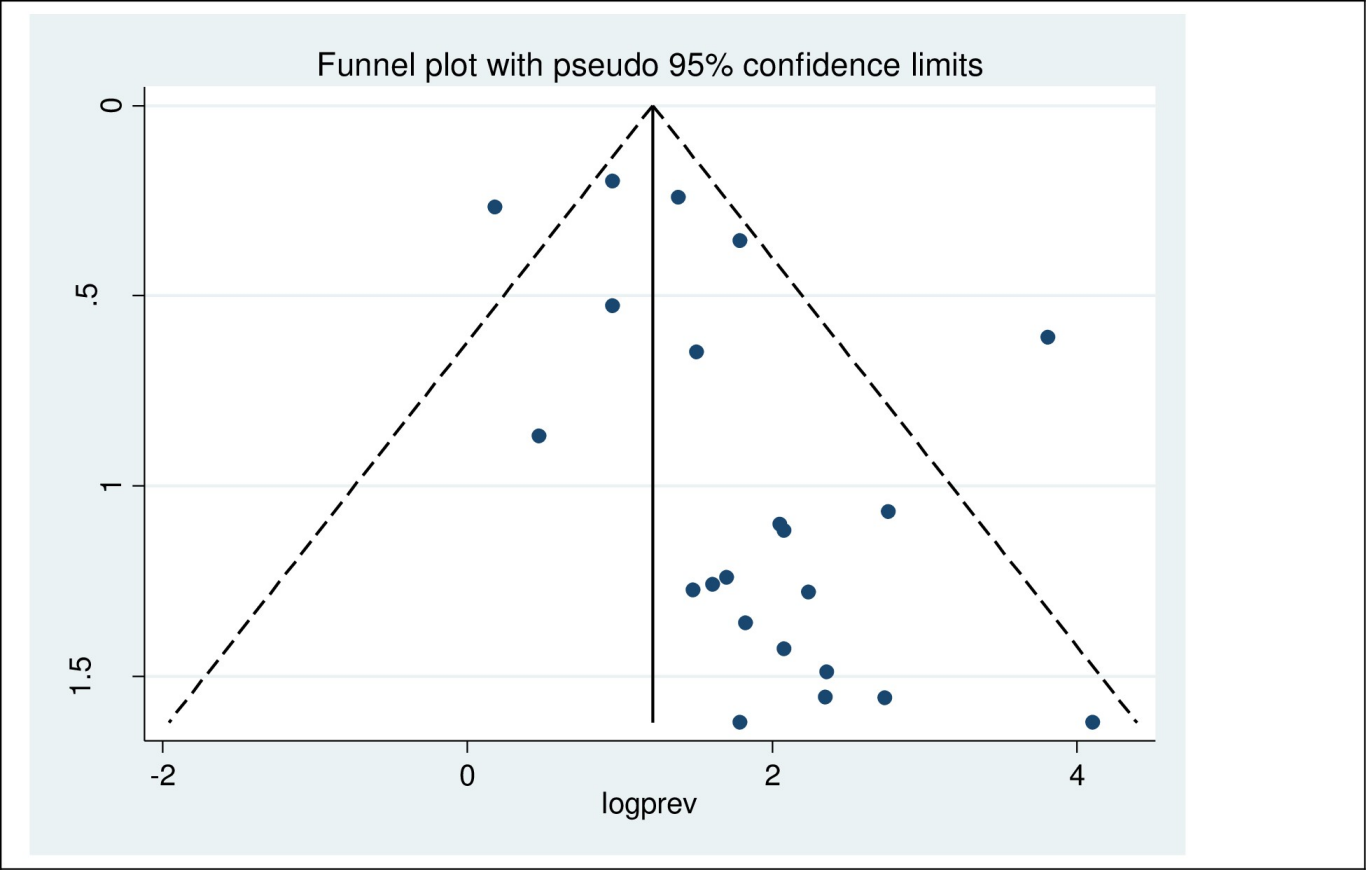
Funnel plot of included studies on the prevalence of HBV among hemodialysis.

**Fig 3:**
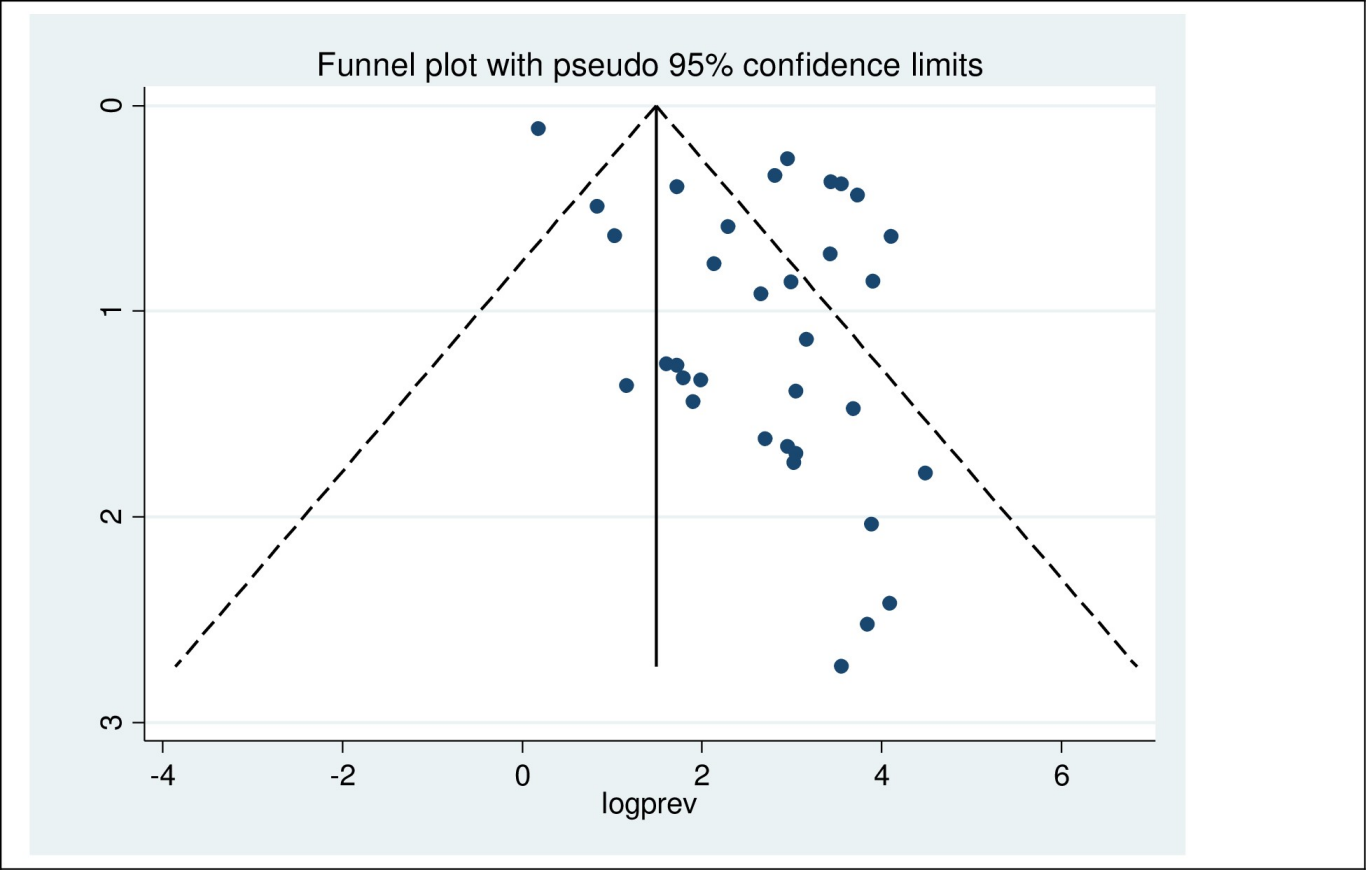
Funnel plot of included studies on the prevalence of HCV among hemodialysis.

The included studies were assessed for potential publication bias statistically by Egger’s test. The result of Egger’s test indicated publication bias, in both HBV (P-values = 0.013) and HCV (P-values = <0.001). This was depicted graphically by a funnel plot which showed a non-symmetrical display of prevalence reported by all the included studies (Figs 2 and 3).

### Association between duration of dialysis and HBV and HCV

**Fig 4:**
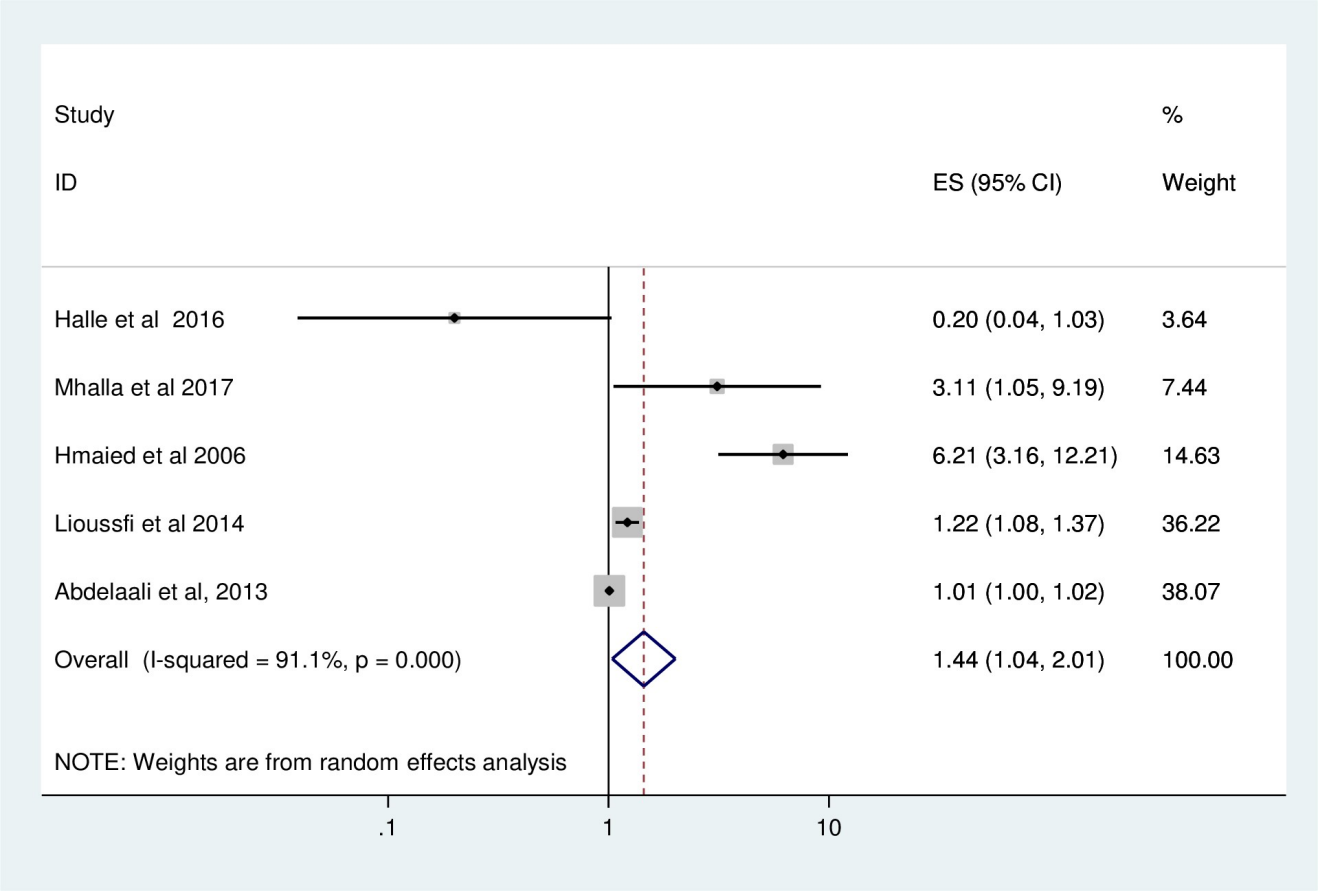
Forest plots which describe associated factor (duration of dialysis) of hepatitis among HD patients in Africa.

Analysis of 5 studies to assess the association between duration of dialysis and HBV and HCV showed that duration of dialysis (OR = 1.44; 95% CI: 1.04, 2.01) was found to be the contributing factor for the occurrence of HBV and HCV among HD patients (Fig 4).

### Sensitivity analysis

**Fig 5:**
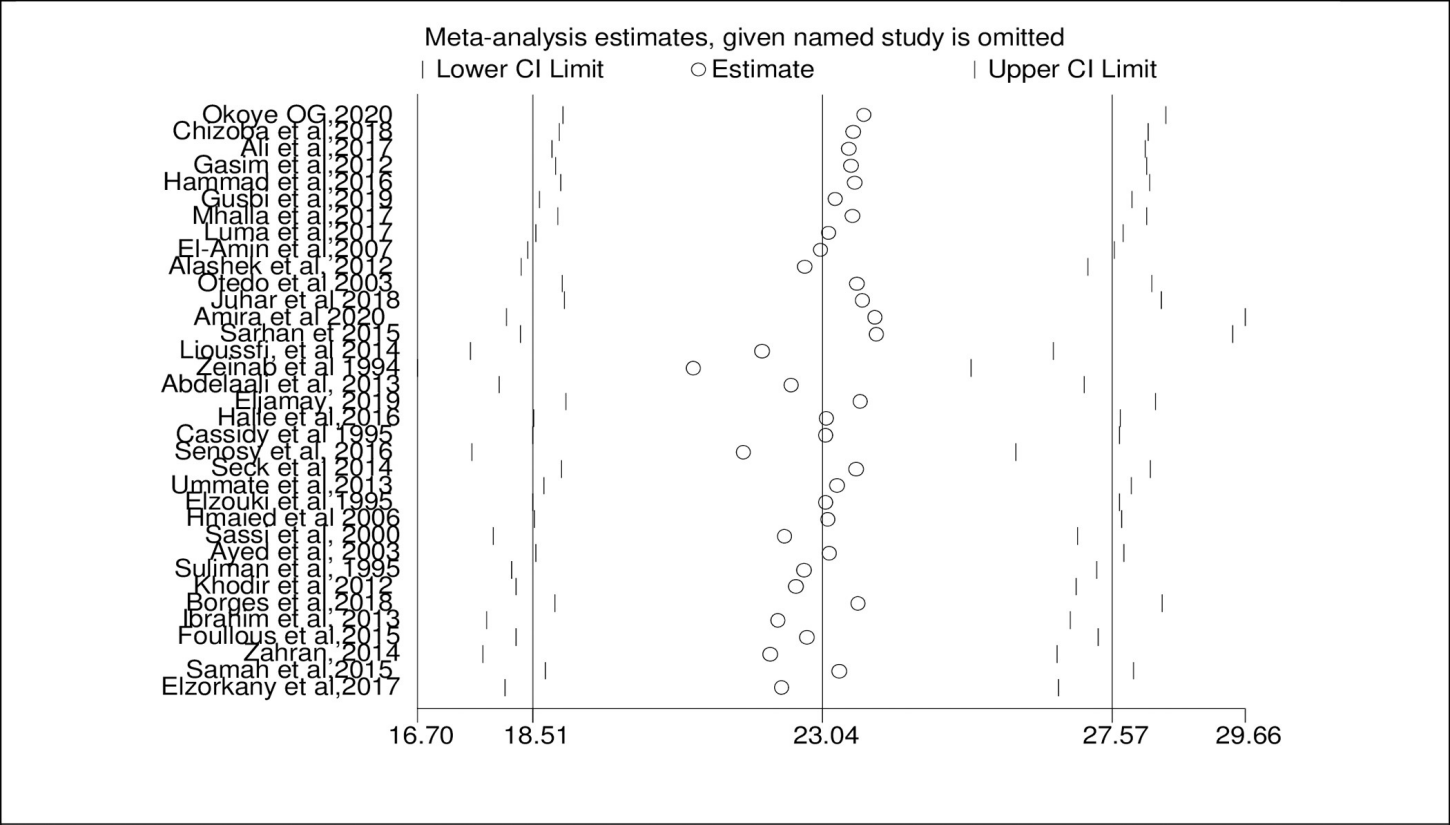
Sensitivity analysis of the included studies to estimate the pooled prevalence of HCV among HD patients.

**Fig 6:**
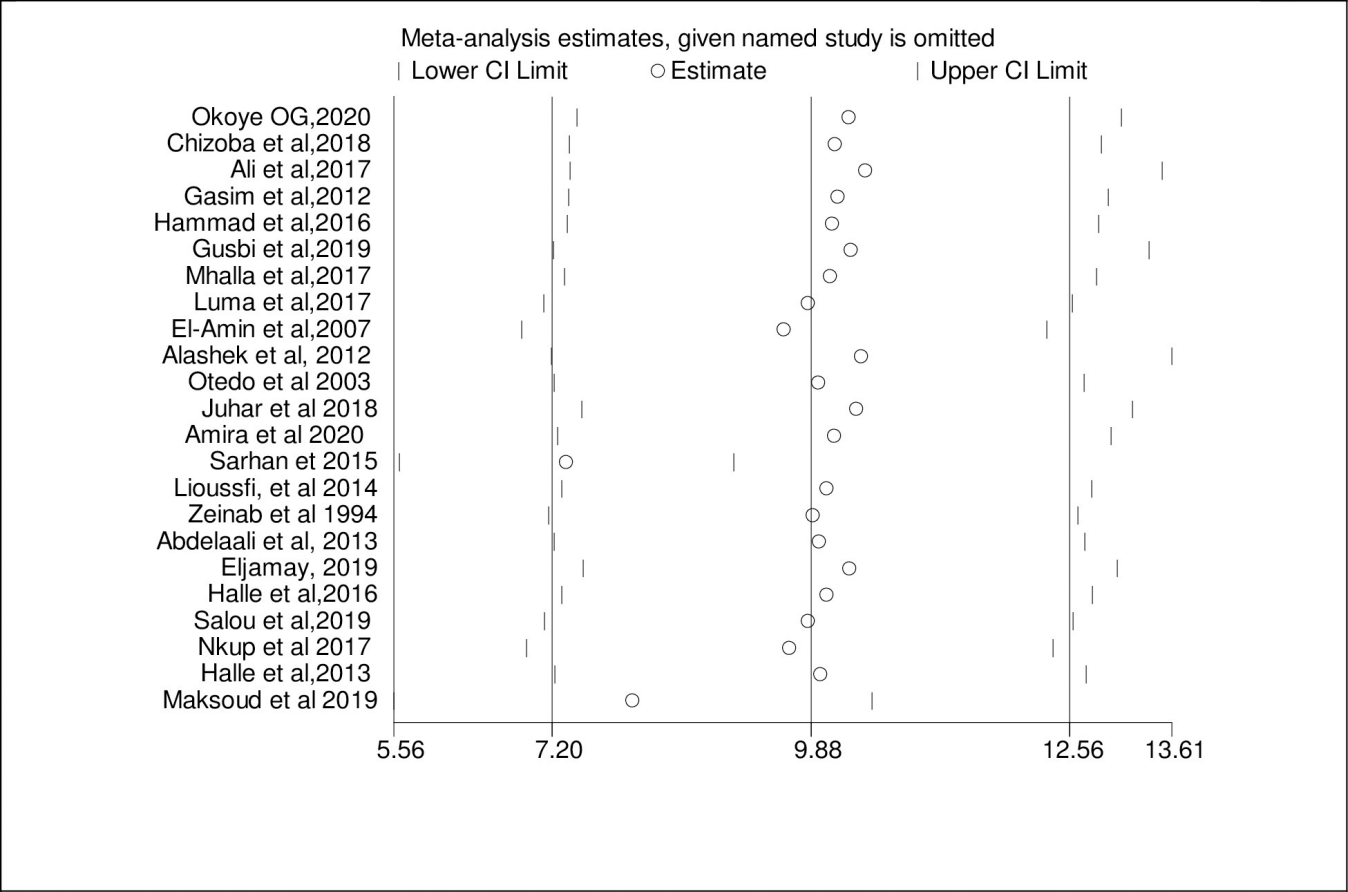
Sensitivity analysis of the included studies to estimate the pooled prevalence of HBV among HD patients.

Due to the high heterogeneity of results in both HCV and HBV, a sensitivity analysis was done by omitting each study step by step to assess the effect of each study on the pooled prevalence. The result showed that omitted studies don’t have a significant effect on the pooled prevalence of HCV and HBV among hemodialysis patients as indicated in Figs 5 and 6, respectively.

#### Meta-regression

In this study, meta-regression were performed on continuous covariates such as study year, the mean age of the participants, sample size, and duration of dialysis. Accordingly, the result of the meta-regression showed that the pooled prevalence of HCV among HD patients was not associated with the study year, the mean age of the participants, and duration of dialysis (Table 4).

**Table 4:** Meta-regression of factors associated with heterogeneity in this study.

Variables	Coefficient	P-value
Mean age	0.091	0.644
Publication year	-0.166	0.276
Sample size	0.005	0.209
Duration of dialysis	-0.07	0.204

## Discussion

This systematic review and meta-analysis was conducted to determine the pooled prevalence of HBV and HCV in HD patients of Africa. Thirty-nine studies based on 23, 538 HD patients were included in this study. The result suggested that the pooled prevalence of HBV and HCV infection among HD patients were 9.88% (95% CI: 7.20–12.56) I^2^ = 97.9% and 23.04% (95% CI: 18.51–2757) I^2^ = 99.6%, respectively. The coinfection of HBV-HCV among HD patients was 7.18% (95% CI: 3.15–11.20) I^2^ = 99.6%. The prevalence of HBV in this study is almost similar to a systematic review conducted in Vietnam by Duong et al [60] which reported that the national pooled prevalence of HBV in the country is 9.7%.

The prevalence of HCV antibody reported in this study (23.04%) is lower than a previous meta-analysis reports in Vietnam (32.6%) [60], Pakistan (32.33%), China (41.1%) [61], and Asia (31%) [62]. On the other hand, the prevalence of HCV infection on HD patients in this study (Africa) is higher than in Europe and the USA (5%-10% or less). This variation in the prevalence rates of HCV across different regions might be because of the disparity of medical conditions in developed and developing countries [63]. Besides, this might be due to a lack of education and awareness of HCV transmission, a lack of qualified and competent medical personnel, a lack of proper health infrastructure, inadequate implementation of practices recommended by the WHO, and inadequate screening of HCV for donated blood [64]. In this systematic review and meta-analysis, the coinfection rate of HBV-HIV was (7.18%). This might support the ideology that declares coinfection rates directly correlate with the burden of individual strain infections.

The subgroup analysis showed that HCV infection prevalence among the HD patients was observed across all regions of Africa. Our results show that the prevalence of HCV among HD patients is higher in Northern Africa (29.69%) than in South Africa (21.00%), in central Africa (14.63%), western Africa (4.37%), and East Africa (3.21%). The prevalence of HBV among HD patients has also been observed across different regions of Africa. Accordingly, the result showed that it was higher in Northern Africa (12.27%), central Africa (7.88%), Western Africa (6.57%), and East Africa (4.09%). This finding was per the fact that North African countries seem to have a higher prevalence than the sub-Saharan and East African countries [31,33,42]. This variability may be due to differences in ethnicity, health provision system, and characteristics of the study population. The prevalence of HCV among HD patients shows a gradual decrement across different categories of study years as it is 12.66% and 11.17% in 1996–2008 and 2016–2020, respectively. A study conducted in Senegal by Seck et al showed that the decline in the prevalence of HCV among HD patients could be due to the development and implementation of preventive strategies, more adherence of the medical staff to the aseptic measures, and a better transfusion policy [44]. Improved infection control, vaccination, and isolation of Hepatitis infected patients during HD are other factors contributing to the low prevalence observed [23].

The prevalence of HBV by ELISA and rapid kits was 7.59 (95%CI: 5.38–9.80) and 7.50% (95% CI: 2.26–12.73), respectively. Similarly, the combined prevalence of HCV in HD patients was 22.55% (95% CI: 17.76–27.34) and 4.46% (95% CI: 1.89–7.23) according to ELISA and rapid kit, respectively. The result of this study highlights that the prevalence of HBV and HCV in HD patients was higher in ELISA as compared to rapid kits. This might be hypothesized that rapid screening test kits are associated with more false negatives compared to the ELISA technique as confirmed by a study conducted in Nigeria [65]. Moreover, a study conducted by Chameera et al says that rapid kits showed less sensitivity and Negative predictive value [66]. However, with several limitations, rapid tests are frequently the only viable option for infectious screening in resource-limited settings [67].

With respect to the associated factors associated with HBV and HCV infection, being on long-term HD was 1.14 more likely to be infected with hepatitis than their counterparts. It is estimated that as the frequency of dialysis increases, the higher the prevalence of hepatitis. There may be other causes of transmission, such as the re-use of the dialyzers and pipelines, frequent cardiopulmonary bypass and repeated needle puncture during the treatment, sharing of dialysis machines, and incomplete disinfection. Control of contagion and minimizing the frequency of blood transfusion are the key to reduce the incidence of hepatitis in HD patients. Moreover, adherence to universal hygiene standards, disinfection of dialysis machines and instruments, avoidance of unnecessary and unsafe injections of blood products, and treatment with antiviral medicines are important to prevent hepatitis [47,61].

Strengths and limitations: the strength of the study is its comprehensive literature search by two independent authors to extract all available published articles. Besides, we included articles published across all corners of Africa (Northern, Central, Western, Eastern, and Southern) that helps to generalize the finding of the study to the continent. The study also comprised on large sample size with a total population of 23,538. The study includes articles published only in English languages that finally compromise the result of the study. There is also significantly high heterogeneity in the included studies.

## Conclusion

This study showed that there is high prevalence of HBV and HCV infections in HD patients in Africa. Therefore, strict adherence to precautions of infection control measures, isolation of seropositive patients, improvement in infrastructures, adequate screening of HBV and HCV for the donated blood, and decentralized HD services is needed to minimize the risk of HBV and HCV infections in HD facilities.

## Supporting information

S1 Table Presents Preferred Reporting Items for Systematic Reviews and Meta-Analyses (PRISMA) checklist. (DOCX)

S2 Table Quality assessment of the studies included in systematic review and meta-analysis of HBV and HCV among HD patients in Africa. (DOCX)

## Abbreviations

ELISA: Enzyme-Linked Immunosorbent Assay
ESRD: End‑Stage Renal Disease
HBV: Hepatitis B virus
HCV: Hepatitis C Virus
HD: Hemodialysis
MEIA: microparticle enzyme immunoassay JBI: Johana bridges institute
RPHA: reverse passive hemagglutination WHO: World Health Organization
