# Supplementary material for: The prevalence and associated factors of hepatitis B and C virus in hemodialysis patients in Africa: A systematic review and meta-analysis
Source: PLoS One. 2021 Jun 22;16(6):e0251570. doi: 10.1371/journal.pone.0251570 (PMC8219139; doi:10.1371/journal.pone.0251570)
Supplement: S2 Table — (DOCX) [file pone.0251570.s002.docx]

| Author, year of publication | Q1 | Q2 | Q3 | Q4 | Q5 | Q6 | Q7 | Q8 | Q9 | Total score (9%) |
| --- | --- | --- | --- | --- | --- | --- | --- | --- | --- | --- |
| Okoye OG,2020 [24] | Y | Y | Y | Y | Y | Y | Y | Y | Y | 9 |
| Chizoba et al,2018 [25] | Y | Y | N | Y | Y | Y | Y | Y | Y | 8 |
| Ali et al,2017 [26] | Y | Y | Y | Y | Y | Y | Y | Y | NA | 8 |
| Gasim et al,2012 [27] | Y | Y | N | N | Y | Y | Y | Y | Y | 7 |
| Hammad et al,2016 [28] | Y | Y | N | Y | Y | Y | Y | Y | NA | 7 |
| Gusbi et al,2019 [29] | N | Y | N | Y | Y | Y | Y | Y | Y | 7 |
| Mhalla et al,2017 [30] | N | Y | Y | Y | Y | Y | Y | Y | Y | 8 |
| Luma et al,2017 [31] | N | Y | N | Y | Y | Y | Y | Y | Y | 7 |
| El-Amin et al,2007 [32] | N | Y | N | N | Y | Y | Y | Y | Y | 6 |
| Alashek et al, 2012 [33] | Y | Y | Y | Y | Y | Y | Y | Y | NA | 8 |
| Otedo et al 2003 [34] | Y | Y | Y | N | Y | Y | Y | Y | NA | 7 |
| Juhar et al 2018 [35] | Y | Y | NA | NA | Y | Y | Y | Y | Y | 7 |
| Amira et al 2020 [36] | Y | Y | Y | Y | Y | Y | NA | Y | Y | 8 |
| Sarhan et 2015 [37] | Y | Y | Y | Y | Y | Y | Y | Y | Y | 9 |
| Lioussfi, et al 2014 [38] | Y | Y | Y | NA | Y | Y | Y | Y | Y | 8 |
| Zeinab et al 1994 [39] | Y | Y | Y | Y | Y | NA | NA | Y | NA | 6 |
| Abdelaali et al, 2013 [40] | Y | Y | Y | NA | Y | Y | Y | Y | Y | 8 |
| Eljamay, 2019 [41] | Y | Y | NA | NA | Y | Y | Y | Y | Y | 7 |
| Halle et al,2016 [42] | Y | Y | Y | Y | Y | Y | Y | Y | Y | 9 |
| Cassidy et al 1995 [43] | Y | Y | N | NA | Y | Y | Y | Y | Y | 7 |
| Senosy et al, 2016 [44] | Y | Y | N | Y | Y | Y | Y | Y | Y | 8 |
| Seck et al 2014 [45] | Y | Y | Y | Y | Y | Y | NA | Y | Y | 8 |
| Ummate et al,2013 [46] | Y | Y | Y | N | Y | Y | Y | Y | Y | 8 |
| Elzouki et al 1995 [47] | Y | Y | Y | NR | Y | Y | Y | Y | Y | 8 |
| Hmaied et al 2006 [48] | Y | Y | N | Y | Y | Y | Y | Y | Y | 8 |
| Sassi et al, 2000 [49] | Y | Y | Y | NA | NA | Y | Y | Y | Y | 7 |
| Ayed et al, 2003 [23] | Y | Y | NA | Y | Y | Y | Y | Y | Y | 8 |
| Suliman et al, 1995 [22] | Y | Y | Y | NA | Y | Y | NA | Y | Y | 7 |
| Khodir et al 2012 [50] | Y | Y | Y | Y | Y | Y | NA | Y | Y | 8 |
| Borges et al,2018 [51] | Y | Y | N | Y | Y | Y | Y | Y | Y | 8 |
| Ibrahim et al, 2013 [52] | Y | Y | Y | Y | Y | Y | Y | Y | Y | 9 |
| Foullous et al,2015 [53] | Y | Y | Y | NA | Y | Y | Y | Y | Y | 8 |
| Zahran, 2014 [54] | Y | Y | Y | NR | Y | Y | Y | Y | Y | 8 |
| Samah et al,2015 [55] | Y | Y | N | Y | Y | Y | N | Y | Y | 7 |
| Elzorkany et al,2017 [56] | Y | Y | Y | N | Y | Y | Y | Y | Y | 8 |
| Salou et al,2019 [57] | Y | Y | N | Y | Y | Y | Y | Y | Y | 8 |
| Nkup et al 2017 [58] | Y | Y | N | Y | Y | Y | NA | Y | Y | 7 |
| Halle et al,2013 [59] | Y | Y | Y | Y | Y | Y | Y | Y | Y | 9 |
| Maksoud et al 2019 [60] | Y | Y | Y | Y | Y | Y | Y | Y | NA | 8 |

Table S2 Quality assessment of the studies included in systematic review and meta-analysis of the prevalence and risk factors of hepatitis B and C virus in hemodialysis patients in Africa: A systematic review and meta-analysis

**Key:** **Y**= Yes; **NR**= Not reported, **NA**=Not appropriate

**Question codes:**

1. Was the sample frame appropriate to address the target population?

2. Were study participants sampled in an appropriate way?

3. Was the sample size adequate?

4. Were the study subjects and the setting described in detail?

5. Was the data analysis conducted with sufficient coverage of the identified sample?

6. Were valid methods used for the identification of the condition?

7. Was the condition measured in a standard, reliable way for all participants?

8. Was there appropriate statistical analysis?

9. was the response rate adequate, and if not, was the low response rate managed appropriately?
